# Spontaneously Conceived Ruptured Heterotopic Pregnancy Presenting with Chest Pain and Dyspnea: A Case Report

**DOI:** 10.5811/cpcem.1671

**Published:** 2024-01-23

**Authors:** Farrah Nasrollahi, Kevin Cao, Janae Hohbein, Wesley Eilbert

**Affiliations:** * University of Illinois, College of Medicine, Department of Emergency Medicine, Chicago, Illinois; † University of Illinois, College of Medicine, Chicago, Illinois

**Keywords:** *heterotopic pregnancy*, *ectopic pregnancy*, *case report*

## Abstract

**Introduction:**

Heterotopic pregnancy, defined as simultaneous intrauterine and ectopic pregnancy, is a rare and potentially life-threatening condition. The incidence of heterotopic pregnancy has significantly increased in the modern era, primarily due to use of assisted reproductive technology. Heterotopic pregnancy in the absence of risk factors is uncommon. The symptoms of heterotopic pregnancy are similar to those of ectopic pregnancy, primarily abdominal pain and vaginal bleeding.

**Case Report:**

We report a case of heterotopic pregnancy occurring in the absence of risk factors and presenting with primary symptoms of chest pain and shortness of breath.

**Conclusion:**

While uncommon, heterotopic pregnancy may occur in patients without risk factors and may present with atypical symptoms such as chest pain and shortness of breath.

Population Health Research Capsule What do we already know about this clinical entity?

*Heterotopic pregnancy (HP) in the absence of risk factors is rare. It typically presents with abdominal pain, vaginal bleeding, and symptoms of hemoperitoneum.*

What makes this presentation of disease reportable?

*A patient with no risk factors for HP presented to the emergency department (ED) with symptoms of pleuritic chest pain and shortness of breath.*

What is the major learning point?

*Heterotopic pregnancy may rarely occur in the absence of risk factors and present with the atypical symptoms of pleuritic chest pain and shortness of breath.*

How might this improve emergency medicine practice?

*
This case of HP occurring in the absence of risk factors and with atypical symptoms may assist clinicians in the diagnosis of similar cases.*

## INTRODUCTION

First described in 1761 as an autopsy finding, heterotopic pregnancy (HP) is a simultaneous intrauterine and ectopic pregnancy. It is a rare and potentially life-threatening condition. The reported incidence of HP in the era before assisted reproductive technology was 1 in 30,000; its incidence has significantly increased in the modern era.^1^ The majority of HPs occur in women with risk factors, with only 29% of HPs occurring in women with no risk factors.^2^ Conception with the use of assisted reproductive technology is the main risk factor for HP.^2^ ^,^ ^3^ Abdominal pain, vaginal bleeding, and symptoms of hemoperitoneum are the main presenting symptoms.^2^ ^–^ ^5^ We report the case of a spontaneously conceived, ruptured HP in a patient who presented with symptoms of pleuritic chest pain and shortness of breath.

## CASE REPORT

A 28-year-old woman at eight weeks gestation based on last menstrual period presented to the emergency department (ED) with three weeks of shortness of breath, pleuritic anterior chest pain, and near syncope with exertion. Associated symptoms included mild lower abdominal cramping and some bright red vaginal spotting. The pregnancy had been conceived naturally, without assisted reproductive technology or hormonal therapy. She had no history of pelvic inflammatory disease and no prior gynecologic surgeries. She had been evaluated at an outside ED one week prior for similar symptoms. Notable workup at the outside ED included a transvaginal ultrasound revealing a live intrauterine pregnancy (IUP) dated at eight weeks gestation with otherwise normal uterus and adnexa. Additional laboratory studies at that time included a serum hemoglobin (Hgb) level of 13.8 grams per deciliter (g/dL) (reference range: 12.0–15.5 g/dL) and a serum beta-human chorionic gonadotropin (bHCG) level of 84,320 milli-international units per milliliter (mIU/mL), an appropriate level for seven weeks gestation.

On presentation to our ED, the patient was afebrile with a pulse of 100 beats per minute and a blood pressure of 106/93 millimeters of mercury. Her oxygen saturation on room air was 99%. On physical examination, her abdomen was soft and without notable tenderness or guarding on palpation. Vaginal speculum exam revealed dark red blood in the vaginal vault, with some blood oozing from a closed cervical os. Bimanual examination was notable for moderate tenderness to palpation of the right adnexa. Laboratory studies included a Hgb level of 11.7 g/dL and bHCG level of 101,505 mIU/ml, an appropriate level for eight weeks gestation.

**Image 1.**
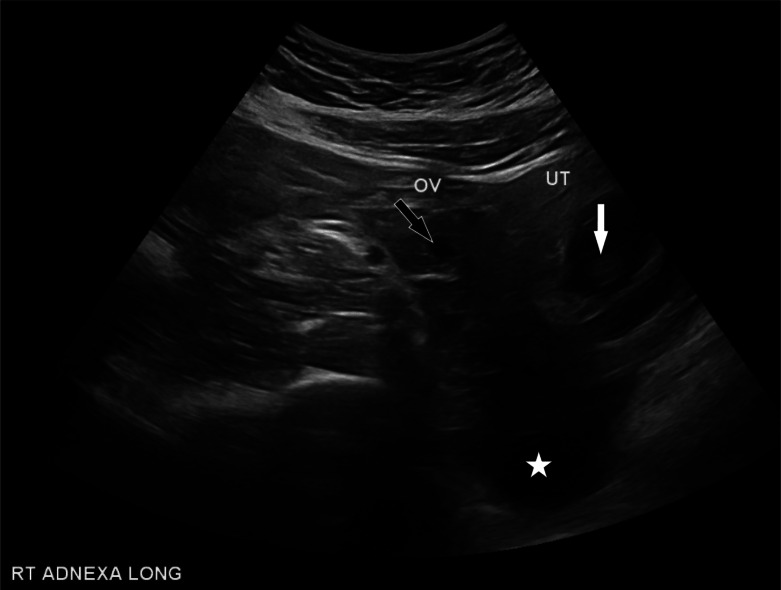
Transvaginal ultrasound showing intrauterine pregnancy (white arrow), right ovary with adjacent cystic structure (black arrow), and fluid in the cul-de-sac (white star). *OV*, ovary; *UT*, uterus.

**Image 2.**
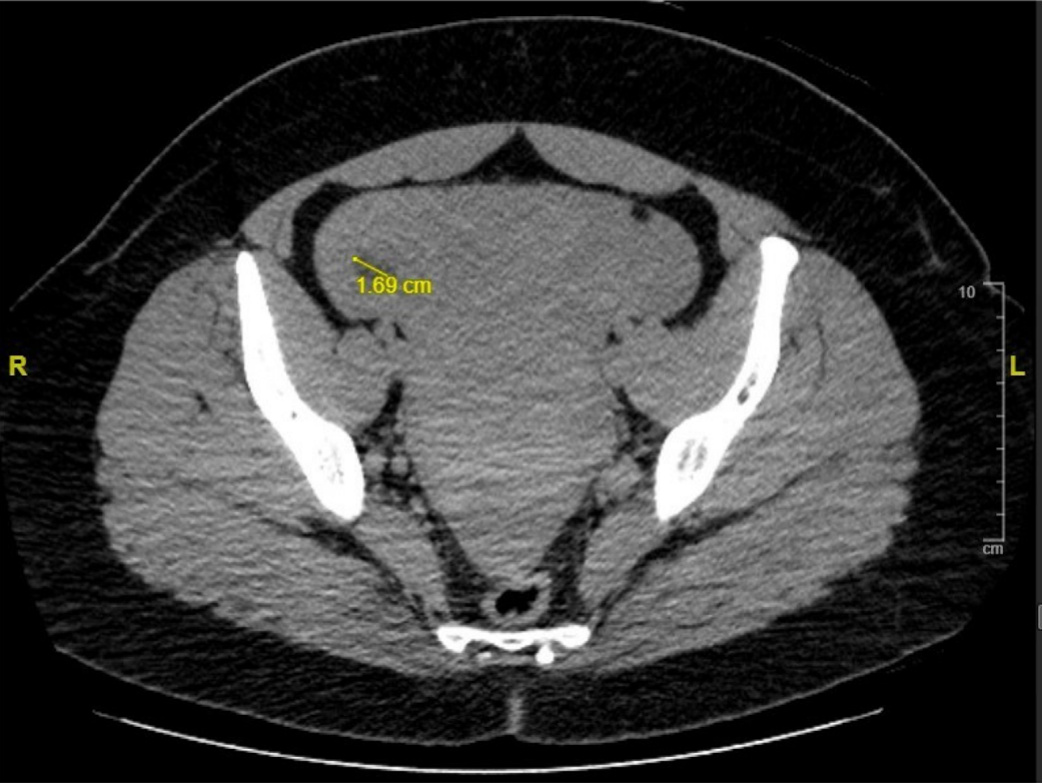
Computed tomography of the pelvis showing a 1.69-centimeter area of low attenuation in the right adnexa. *cm*, centimeter.

Transvaginal ultrasound showed a live IUP with an estimated gestational age of eight weeks. The left ovary was not visualized, and the right ovary was noted to be normal in size and morphology with a thick-walled cystic structure thought to represent a corpus luteum cyst (Image 1). A moderate amount of hypoechoic material was noted in the cul-de-sac consistent with intraperitoneal blood. Computed tomography (CT) pulmonary angiography was obtained due to concern for pulmonary embolism. While no pulmonary embolism was seen on the CT, free fluid was seen incidentally in the peritoneum. After discussion with radiology and obstetrics as well as risks and benefits discussed with the patient, a CT of the abdomen and pelvis was obtained to rule out a nonpelvic source of hemoperitoneum. The CT redemonstrated moderate hemoperitoneum of suspected pelvic origin and a 1.69-centimeter area of nonspecific low attenuation in the right adnexa (Image 2). The patient was admitted for serial abdominal examinations and Hgb levels.

On hospital day three, the patient’s Hgb level dropped to 8.3 g/dL, and she was taken for exploratory laparotomy. A ruptured right fallopian tube with ectopic pregnancy was found. The patient underwent a right salpingectomy, and after an unremarkable postoperative course was discharged home. The patient ultimately delivered a healthy infant at term.

## DISCUSSION

While rare, the incidence of HP has increased significantly in the last 50 years with the increased use of assisted reproductive technology.^6^ The risk of HP among women who have undergone in vitro fertilization is estimated to be as high as one in 100.^7^ While assisted reproductive technology is the main risk factor for HP, the presence of any traditional risk factors for ectopic pregnancy also increases the likelihood of HP (Table). Previous authors have recommended that further investigation of HP is not necessary in cases where an IUP is identified on point of care ultrasound in the ED and there are no risk factors for HP.^8^ This case, along with others reporting HP in the absence of risk factors, suggests this practice is not foolproof, particularly if free intraperitoneal fluid is noted on the ultrasound.^9^

**Table.** Risk factors for heterotopic pregnancy.

• Assisted reproductive technology including in vitro fertilization and fertility medication
• History of pelvic inflammatory disease
• Prior pelvic or abdominal surgeries
• Endometriosis
• Previous use of an intrauterine contraceptive device
• Previous ectopic pregnancy

As with this case, the majority (71%) of HPs are diagnosed between 5-8 weeks gestation, with 29% diagnosed after the ninth week.^3^ With abdominal pain and vaginal bleeding as the primary symptoms of HP, it is likely that many HPs are initially misdiagnosed as threatened abortion, as in this case. In fact, 33% of HPs have a previously documented IUP at the time of diagnosis.^2^ Further adding to the diagnostic challenge of HP is the fact that 5% of pregnancies will have an associated adnexal mass, with corpus luteum cysts being one of the main causes.^10^ Similar to this case, it is likely that many heterotopic pregnancies are initially misdiagnosed as an IUP with an associated hemorrhagic corpus luteum cyst. Serial bHCG measurements are not a useful aid in the diagnosis of HP due to the concomitant IUP.^2^ ^,^ ^6^ While in the past the diagnosis of HP was made at the time of surgery for most cases, approximately 66% of cases are now being diagnosed by ultrasound.^2^ Transvaginal ultrasound is the imaging modality of choice for HP, and magnetic resonance imaging may be used to provide additional information without the use of ionizing radiation.

To our knowledge, only one case of heterotopic pregnancy and two cases of ectopic pregnancy presenting with chest pain have been previously described.^11^ ^–^ ^13^ As with our case, in each of these three previously reported cases pulmonary embolism was an initial concern. It is likely the chest pain described in all these cases was due to diaphragmatic irritation from intraperitoneal blood causing referred pain.

The lack of significant tachycardia in this patient despite the presence of significant hemoperitoneum has been previously described.^14^ ^,^ ^15^ It is theorized that this phenomenon may be due to pregnant patients typically being young and healthy and, therefore, less likely to develop an early tachycardic response to blood loss.^14^ Another theory is that blood in the peritoneum may trigger a parasympathetic reflex mediated by the vagus or pelvic nerves.^15^

The ultimate goal of the management of HP is to terminate the extrauterine pregnancy while minimizing the threat to the IUP. Treatment options include expectant management, sonographic-guided embryo aspiration with or without embryo toxic drugs, and surgical intervention.^6^ The chosen treatment approach for HP is dependent on several factors, including the location of the extrauterine pregnancy and the hemodynamic stability of the patient.^6^ As with this case, patients with significant intraperitoneal hemorrhage should be managed surgically.^4^ Recent case series have reported 74–88% of HPs will result in a live birth.^2^ ^,^ ^4^ ^,^ ^5^

## CONCLUSION

While uncommon, HP may occur in patients without associated risk factors. Heterotopic pregnancy typically presents with abdominal pain and vaginal bleeding and is often initially misdiagnosed as threatened abortion. As this case illustrates, HP may present with chief complaints of dyspnea and chest pain, resulting from intraperitoneal hemorrhage and diaphragmatic irritation.
